# The synergistic effects of anoikis-related genes and EMT-related genes in the prognostic prediction of Wilms tumor

**DOI:** 10.3389/fmolb.2024.1469775

**Published:** 2024-09-16

**Authors:** Kexin Meng, Zerui Zhao, Yaqing Gao, Keliang Wu, Wei Liu, Xiaoqing Wang, Yi Zheng, Wei Zhao, Bei Wang

**Affiliations:** ^1^ Department of Medical Ultrasound, The First Affiliated Hospital of Shandong First Medical University and Shandong Provincial Qianfoshan Hospital, Shandong Medical and Health Key Laboratory of Abdominal Medical Imaging, Jinan, China; ^2^ Department of Clinical Pharmacy, Clinical Trial Center, The First Affiliated Hospital of Shandong First Medical University and Shandong Provincial Qianfoshan Hospital, Shandong Engineering and Technology Research Center for Pediatric Drug Development, Medicine and Health Key Laboratory of Clinical Pharmacy, Jinan, China; ^3^ Department of Clinical Pharmacy, School of Pharmaceutical Sciences, Shandong University, Jinan, China; ^4^ Department of Pediatric Surgery, Shandong Provincial Hospital Affiliated to Shandong First Medical University, Jinan, China

**Keywords:** Wilms tumor, anoikis, EMT, prognostic model, tumor microenvironment

## Abstract

Wilms tumor (WT) is the most common type of malignant abdominal tumor in children; it exhibits a high degree of malignancy, grow rapidly, and is prone to metastasis. This study aimed to construct a prognosis model based on anoikis-related genes (ARGs) and epithelial-mesenchymal transition (EMT)-related genes (ERGs) for WT patients; we assessed the characteristics of the tumor microenvironment and treatment efficacy, as well as identifying potential therapeutic targets. To this end, we downloaded transcriptome sequencing data and clinical data for WT and normal renal cortices and used R to construct and validate the prognostic model based on ARGs and ERGs. Additionally, we performed clinical feature analysis, nomogram construction, mutation analysis, drug sensitivity analysis, Connectivity Map (cMAP) analysis, functional enrichment analysis, and immune infiltration analysis. Finally, we screened the hub gene using the STRING database and validated it via experiments. In this way, we constructed a model with good accuracy and robustness, which was composed of seven anoikis- and EMT-related genes. Paclitaxel and mesna were selected as potential chemotherapeutic drugs and adjuvant chemotherapeutic drugs for the WT high-risk group by using the Genomics of Drug Sensitivity in Cancer (GDSC) and cMAP compound libraries, respectively. We proved the existence of a strong correlation between invasive immune cells and prognostic genes and risk scores. Next, we selected NTRK2 as the hub gene, and *in vitro* experiments confirmed that its inhibition can significantly inhibit the proliferation and migration of tumor cells and promote late apoptosis. In summary, we screened out the potential biomarkers and chemotherapeutic drugs that can improve the prognosis of patients with WT.

## 1 Introduction

Wilms tumor (WT), also known as nephroblastoma, is a common type of embryonal childhood tumor; it is also one of the predominant types of childhood kidney cancer (Davidoff, 2012). They account for about 90% of childhood renal tumors and 7% of childhood cancers (Breslow et al., 1993). WT is thought to be caused by aberrant renal development (Rivera and Haber, 2005). Although the prognosis for WT patients is good, 13% of WT patients experience a two-year relapse after their tumor diagnosis (Furtwangler et al., 2011). To improve the risk assessment and therapy stratification of WT, it is essential to explore the main mechanism of its occurrence and development (Liu et al., 2020).

Anoikis arises due to the rupture of cell‒cell or cell and extracellular matrix attachments, leading to a specific form of programmed apoptosis, which helps to maintain tissue homeostasis by eliminating misplaced or dislodged cells (Han et al., 2021). Anoikis was first described in epithelial and endothelial cells and was found to be an important mechanism of cancer invasion and metastasis (Kakavandi et al., 2018). The onset of anoikis resistance can help detached cells to circumvent death signaling pathways, allowing cells to survive under unfavorable conditions (Adeshakin et al., 2021; Di Micco et al., 2021). Epithelial-mesenchymal transition (EMT) refers to the process by which cells change from epithelioid to mesenchymal. It is often accompanied by changes in cell morphology, loss of polarity, increased invasiveness, resistance to anoikis, and secretion of extracellular matrix (Huang et al., 2020). The importance of EMT programs in tumor progression has been established in the past two decades (Huang et al., 2013), with a rapidly growing number of studies demonstrating the activation of EMT programs during the process of malignant progression (Ye and Weinberg, 2015; Shibue and Weinberg, 2017; Nieto et al., 2016). The contributions of the EMT program to tumor cell phenotypes have been most intensively studied in relation to carcinomas (Lambert et al., 2017; Thiery, 2002). At present, a large number of reports have proved that: EMT allows cancer cells to detach from neighboring cells, overcome anoikis, and migrate from their primary location to metastatic sites. Furthermore, anoikis and EMT may share some signaling pathways and regulatory molecules (Sakamoto and Kyprianou, 2010), they undergo a few instances of crosstalk (Cao et al., 2016), for example, the development of anoikis resistance and EMT of lung cancer cells can be restrained via suppressing JAK2/STAT3 and SHP2/Grb2/PI3K/AKT signaling cascades (Wang et al., 2022a); The prognostic risk model of colon adenocarcinoma suggests that a positive correlation among anoikis resistance, EMT, and liver/lung metastasis of colon adenocarcinoma (Zhou et al., 2023); In the metastasis of cervical cancer, Nrf2 plays a crucial role, which can enhance EMT and resistance to anoikis by promoting the expression of Snail1 (Zhang et al., 2023). In addition, some studies have separately evaluated the implications of anoikis and EMT in WT (Giner et al., 2011; Guo et al., 2022), both of them play a non-negligible role in tumorigenesis, tumor invasion, and tumor infiltration. Therefore, the co-analysis of EMT and anoikis is vital in relation to WT (Amoedo et al., 2014). However, the current research on anoikis and the EMT pathway in WT still lacks a large-sample systematic analysis and clinical models with good practicability and robustness. The process of our study is shown in Supplementary Figure S1.

In this study, we systematically demonstrated the importance of the misalignment and crosstalk of anoikis pathway and EMT pathway in WT patients in tumorigenesis and development. We constructed a risk model composed of seven risk factors for anoikis-related genes (ARGs) and EMT-related genes (ERGs) in order to predict the prognosis of WT patients; we then verified its reliability and robustness using a training cohort and test cohort. In addition, we discussed the predictive effect of the risk model on the sensitivity of commonly used chemotherapeutic drugs and screened out potential drugs that might improve the clinical treatment of patients in the high-risk group. Then, we associated the risk model with tumor microenvironment (TME) and analyzed the differential genes between the high- and low-risk groups to explore the regulatory mechanism behind them using Kyoto Encyclopedia of Genes and Genomes (KEGG) enrichment. Finally, the hub gene with the inherent potential to become a prognostic biomarker and a drug target was screened and verified using clinical samples and *in vitro* experiments.

## 2 Materials and methods

### 2.1 Data collection

We obtained childhood Wilms tumor patient samples from the Therapeutically Applicable Research to Generate Effective Treatments (TARGET) database (https://www.cancer.gov/ccg/research/genome-sequencing/target) and Genotype-Tissue Expression (GTEx) database (https://www.genome.gov/Funded-Programs-Projects/Genotype-Tissue-Expression-Project). We used sequencing data and clinical data of normal renal cortex tissue and normal adjacent tissues as the training set (Grossman et al., 2016). The “sva” package’s ComBat_seq algorithm was used to remove batch effects between tumor and normal tissue (Leek et al., 2012). The clinical data included patient survival time, survival status, age, gender, stage, etc., and we excluded patients with incomplete information. We also downloaded the gene expression data and corresponding clinical data from the Genomic Data Commons (GDC) (https://portal.gdc.cancer.gov/) as the testing set. Additionally, we downloaded 198 genes from the Molecular Signatures Database (MSigDB) (https://www.gsea-msigdb.org/gsea/msigdb) as EMT-related genes and derived human anoikis-associated genes from GeneCards (https://www.genecards.org/) (Subramanian et al., 2005; Stelzer et al., 2016).

### 2.2 Pathway scoring of anoikis and EMT

First, pathway scoring was calculated using the GSVA algorithm for the ARGs (consisting of 408 relevant genes) and the ERGs (comprising 174 relevant genes) (Hanzelmann et al., 2013). Subsequently, the Wilcoxon rank-sum test was employed to assess the differences between tumor and normal tissues.

### 2.3 Differential analysis of ARG and ERG expression

Using the “limma” package (Ritchie et al., 2015), “DESeq2” package (Love et al., 2014), “edgeR” package (Chen et al., 2016), and Wilcoxon test in R, we calculated the differentially expressed genes (DEGs) between the WT samples and normal samples. The DEGs were selected based on the criteria of |logFC| > 1 and *p*-value < 0.05. The intersection of DEGs obtained from the “limma,” “DESeq2,” and “edgeR” packages was taken. The visualization of the results was carried out using the “ggplot2” and “tinyarray” packages (Valero-Mora, 2010). The “venn” package was used to intersect the DEGs, ARGs, and ERGs (Bardou et al., 2014).

### 2.4 Construction and validation of the prognostic model of ARGs and ERGs

In the training cohort, the “survival” package (Therneau et al., 2000) was used to perform a univariate Cox analysis on the ARGs and ERGs, resulting in the identification of genes where the expression values were significantly associated with overall survival (*p* < 0.05). Subsequently, a penalty-based least absolute shrinkage and selection operator (Lasso) logistic model was established to identify the candidate prognostic genes (Friedman et al., 2010). Furthermore, the Akaike Information Criterion (AIC) method was applied in the multiple Cox regression analysis, using the derived regression coefficients in combination with the linear integration of the expression levels of the selected relevant genes to establish the optimal risk model. The risk score was calculated using the following equation:

$$\text{Risk score}=\sum_{i=1}^{N}\left({\text{Coef}}_{i}\times {\mathrm{Exp}}_{i}\right)$$

where Exp_i_ represents the expression values of the relevant genes, and Coef_i_ represents the corresponding regression coefficients calculated through multiple Cox regression analysis. Additionally, MP2PRT data are used as the validation cohort (Gadd et al., 2022).

### 2.5 Clinical feature analysis

Utilizing the “ggstatsplot” package, we compared the differences in the risk scores between different clinical feature groups and the expression patterns of risk factors (Patil, 2021). Subsequently, we analyzed the sample information for different first events within the high-risk and low-risk groups.

### 2.6 Construction of the nomogram

The “RMS” package and the “survival” package were used to construct a nomogram based on patients’ clinical and pathological variables, including age, gender, stage, classification, and the risk score based on the risk factors (Nunez et al., 2011). This nomogram helps to create a personalized predictive model. To assess the accuracy of the nomogram in predicting the 1-year, 3-year, and 5-year survival rates for WT patients, calibration curves and decision curve analysis (DCA) were employed (Rousson and Zumbrunn, 2011). Then, the “survival” package, in conjunction with the “ROC” package, was used to create Kaplan–Meier survival curves and receiver operating characteristic (ROC) curves based on the risk scores calculated using the nomogram. These curves help in visualizing and evaluating survival outcomes and the performance of the risk score in predicting survival.

### 2.7 Gene mutation analysis

First, we obtained the mutation data of patients with Wilms tumor from the Genomic Data Commons (https://portal.gdc.cancer.gov/). Next, we calculated the mutation frequency of the top 20 genes using the “maftools” package and visualized them using the “oncoplot” function (Mayakonda et al., 2018). Subsequently, we calculated the tumor mutation burden (TMB) for all tumor samples and assessed the distribution differences of the TMB values in the different risk groups using the Wilcoxon rank-sum test, visualizing the results using violin plots.

### 2.8 Prediction of drug sensitivity and immunotherapy responses

Using the Genomics of Drug Sensitivity in Cancer (GDSC) database (https://www.cancerrxgene.org/), we downloaded gene expression data for all 805 cell lines from the GDSC2 dataset to create a training set (Yang et al., 2013). Subsequently, we employed the “oncoPredict” package to build a model for assessing the sensitivity of WT patients in the different risk groups to chemotherapy drugs (Maeser et al., 2021). We used the Tumor Immune Dysfunction and Exclusion (TIDE) tool (https://tide.dfci.harvard.edu/) to evaluate the effectiveness of immunotherapy and the potential for immune escape in patients (Fu et al., 2020). A higher TIDE score indicates poorer responses to immunotherapy and a greater likelihood of immune escape. Finally, we conducted Wilcoxon rank-sum tests to assess the differences in the drug sensitivity and immunotherapy response between the different risk groups.

### 2.9 Functional enrichment analysis

We performed differential analysis between the high-risk and low-risk groups and conducted pathway enrichment analysis on the differentially expressed genes based on KEGG gene sets (Kanehisa et al., 2017).

### 2.10 Immune infiltration analysis

The single-sample Gene Set Enrichment Analysis (ssGSEA) algorithm from the “GSVA” package was used to assess the abundance of different immune cell subtypes in the tumor microenvironment based on the risk score of individual tumor samples (Charoentong et al., 2017).

### 2.11 Protein–protein interaction network

We utilized the STRING database (https://cn.string-db.org/) to construct a protein–protein interaction network for the model genes and used the MCC algorithm to identify the hub gene of the risk model (https://cytoscape.org/) (Szklarczyk et al., 2011).

### 2.12 Patient tissue specimens and cell lines

The tissue specimens (including tumor and normal adjacent tissues) of 12 WT patients were extracted from the patients after surgical resections in the Shandong Provincial Hospital Affiliated to Shandong First Medical University (SPH). Informed consent was obtained from the patients before inclusion in the study. The studies involving humans were approved by the Ethics Committee of Shandong Provincial Hospital Affiliated to Shandong First Medical University (ethics approval number: SZRJJ:NO.2021–140) and complied with all relevant ethical regulations for clinical study. All specimens were sampled within 10 min after resections and subsequently extracted total RNA for transcriptome sequencing or fixed in 10% formalin. The ethics committee in the hospital approved the experimental procedures used in the study, and the patients were also asked to sign a consent form. The 17.94 Wilms tumor cell lines were supplied by the Leibniz institute (DSMZ-German Collection of Microorganisms and Cell Cultures GmbH) and the HEK293 cell lines were sourced from the Chinese Academy of Sciences Cell Bank. These cells were cultured in a DMEM medium (Hyclone, United States) containing 10% fetal bovine serum (Gibco, United States), and 1% penicillin/streptomycin solution (Beyotime, China), at 37°C, under 5% CO_2_ and 95% humidity conditions. All the experiments were conducted using mycoplasma-free cells.

### 2.13 Western blotting

WT clinical tissues (including tumor and normal adjacent tissues) and two cell lines (17.94 and HEK293) were used for the WB analysis. Tissues and cells were lysed with a RIPA lysis buffer supplemented with 1% phenylmethanesulfonyl fluoride (Solarbio). Then, the protein concentration was measured using a BCA Protein Assay Kit (Beyotime). The lysates were fractionated using SDS-PAGE and the isolates were transferred to PVDF membranes (Millipore, IPVH00010, NH, United States). The blots were probed with specific primary antibodies followed by a secondary antibody, and the membranes were then detected using ECL (Sigma, WBULS0500, MO, United States). TRKB (ab134155: 1:10,000) and GAPDH (ab8245; 1:10,000) antibodies were purchased from Abcam. Secondary antibodies were conjugated with HRP (Proteintech; PR30009; 1:10,000).

### 2.14 IHC analysis

The expression of TRKB in WT tissues were evaluated via an immunohistochemical analysis. Paraffin-embedded tissues were cut into 4 mm sections. Sections were deparaffinized and boiled in 10 mM citrate buffer (pH 6.0) for antigen retrieval, and 3% H_2_O_2_ was used to block endogenous peroxidase activity. TRKB (Abcam ab134155 1:100) antibodies were used as primary antibodies.

### 2.15 CCK8 assay

The 17.94 cells were seeded in the 96-well plate at a density of 1.5 × 10^4^ cells per well. After the cells adhered to the wall, they were cultured with eight different concentrations of ANA-12 (0, 1, 2, 5, 10, 20, 30, and 40 μM). After culturing for 24 h or 48h, 10μL/well CCK8 (Biosharp) was added. This was followed by continuous incubation for 1 h, and the absorbance was measured at 450 nm. All experiments were executed in triplicate.

### 2.16 Flow cytometry

The 17.94 cells were cultured for 24 h with a medium containing various concentrations of ANA-12 (0, 10, 20, and 40 μM). Then, they were dissociated using trypsin without EDTA. After washing three times with precooled PBS, the cells were diluted in 100 μL binding buffer. Then, we detected cell apoptosis using an Annexin V-APC/7-AAD apoptosis kit (MULTI SCIENCES). Then, 5 μL Annexin V-PE and 10 μL 7-AAD were added into the above binding buffer. After incubation for 10 min at room temperature, 400 μL binding buffer was added. Finally, the cell apoptosis state was analyzed via flow cytometry. All the experiments were performed in triplicate.

### 2.17 Wound healing assay

The 17.94 cells were added to 6-well plates to create a confluent monolayer. Linear scratches were made using a 10 µL pipette tip. The ANA-12 (Selleck) was dissolved in dimethyl sulfoxide (DMSO, Sigma Aldrich), and then the solution was diluted in DMEM to three concentrations (10μM, 20μM, and 40 μM) and added to the corresponding well. The migration of cells into the scratched areas was monitored and photographed after 24 h. The results were analyzed using ImageJ.

### 2.18 Transwell assay

Transwell chambers with Matrigel-coated membranes were used to assess the invasion potential of the 17.94 cells. The 17.94 cells were seeded in the upper chamber supplemented with a serum-free medium, and a medium containing different concentrations of ANA-12 was added (0 μM, 10, 20, and 40 μM); meanwhile, the lower chamber contained a medium with 20% FBS. After the 17.94 cells were incubated for 24 h at 37°C, nonmigrating cells were removed with cotton swabs. Migrated or invaded cells on the bottom of the membrane were fixed with 4% paraformaldehyde for 15 min and stained with crystal violet for 15 min. Then, stained cells were assessed via counting under a microscope.

## 3 Results

### 3.1 The differential expression of anoikis-associated genes and EMT-related genes in WT patients

**FIGURE 1:**
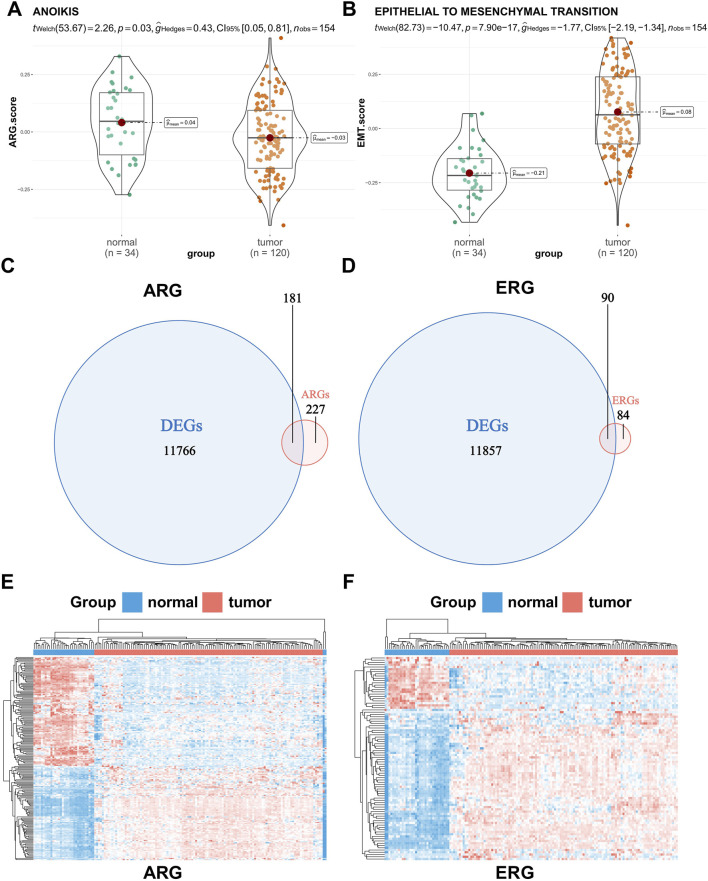
Difference analysis of the pathway score and gene expression between normal and tumor tissues. **(A,B)** Difference in the anoikis and epithelial-mesenchymal transition (EMT) pathway score between normal and tumor tissues. **(C,D)** Venn plots of the intersection of anoikis-related genes (ARGs) or EMT-related genes (ERGs) and differentially expressed genes between normal and tumor tissues. **(E,F)** Hierarchical clustering of differentially expressed genes between normal and tumor tissues (red represents a tumor sample or gene up-regulation, blue represents a normal sample or gene down-regulation).

First, 408 human anoikis-related genes were downloaded from GeneCards. Then, 174 pathway genes were downloaded as EMT-related genes from the Molecular Signature Database (MsigDB). We used the GSVA algorithm to calculate pathway scores based on the anoikis gene set (comprising 408 related genes) and the EMT gene set (comprising 174 related genes), and then employed Wilcoxon rank-sum tests to assess the differences between tumor and normal tissues (Figures 1A, B). Our findings revealed that tumor tissues tend to inhibit anoikis but activate EMT, thereby promoting the occurrence and development of the tumor. In order to further demonstrate the accuracy of analysis results, we used seven pairs of matched clinically-collected transcriptome data of Wilms tumor and adjacent tissue samples for verification. The results showed that the verification results of our SPH cohort were consistent with the trend of previous analysis results (Supplementary Figures S2A, B). To explore whether these genes can accurately distinguish WT patient samples from normal samples, we conducted differential expression analysis on 120 tumor tissues and six normal adjacent tissues samples from the TARGET-WT dataset and 28 normal renal cortex samples from the GTEx database. Among these genes, 11,947 showed significant differential expression (*p* < 0.05 and |logFC| ≥ 1). Subsequently, the intersection of anoikis-associated genes, EMT-related genes, and differentially expressed genes yielded 181 anoikic differentially expressed genes and 90 EMT differentially expressed genes (Figures 1C, D). In the heatmap, hierarchical clustering based on differential genes clearly shows the general situation of genomic differences between normal and tumor tissues (Figures 1E, F).

### 3.2 Construction and validation of the anoikis and EMT gene signature

**FIGURE 2:**
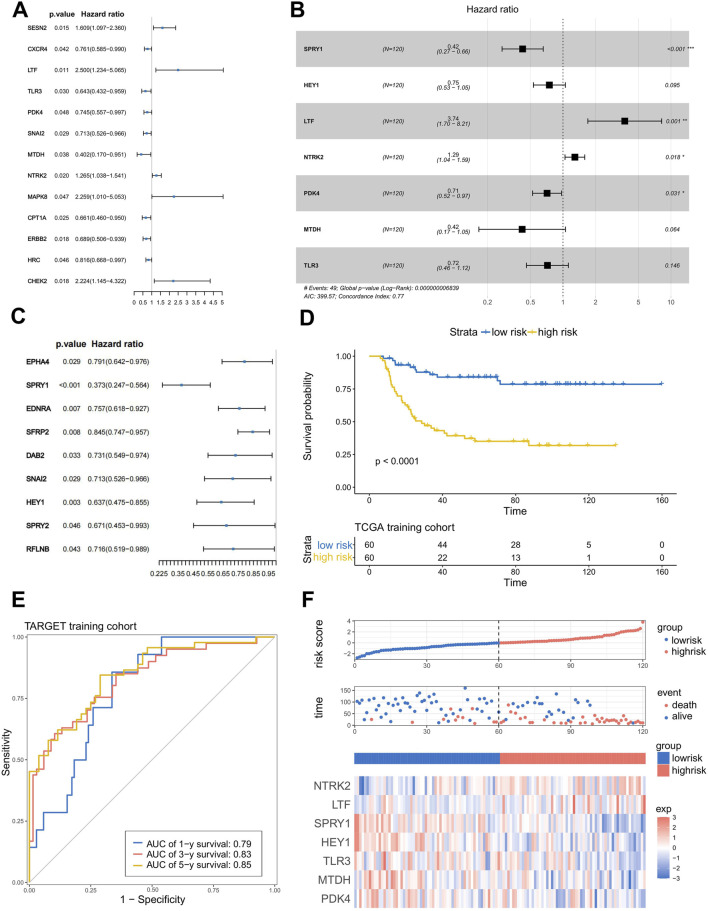
Construction and verification of the anoikis- and EMT-related prognostic model. **(A,C)** Results of the univariate Cox regression analysis of ARG and ERG. **(B)** Seven genes were chosen to establish a prognostic model. **(D)** K–M curve of the prognostic model in the TARGET training cohort (Log-rank test). **(E)** ROC curves of the prognostic model for predicting the 1-, 3-, and 5-year OS times in the TARGET training cohort. **(F)** Risk score distribution plots, survival status plots, and risk factors heatmap of the TARGET training cohort.

After confirming the profound and complex impact of ARGs and ERGs on WT patients, we aimed to construct a clinical model with good accuracy and robustness to assist in clinical diagnosis and treatment. First, in the training set, based on the anoikis-related differentially expressed genes and EMT-related differentially expressed genes obtained above, we performed univariate Cox regression to screen for prognostically significant genes (*P* < 0.05) and created forest plots of risk, where most genes were categorized as risk genes (HR > 1). In the anoikis category, a total of 13 different genes showed prognostic differences (Figure 2A). In the EMT category, nine different genes exhibited prognostic differences (Figure 2C). Then, LASSO analysis was applied to obtain the hub genes in the two groups of overlapped genes (Supplementary Figures S3A, B). Subsequently, we merged the LASSO filtering results of the ARGs and ERGs, inputted them into a multifactorial Cox stepwise regression, and created the prognostic model. This process led to the identification of seven independent prognostic genes (NTRK2, SPRY1, HEY1, LTF, PDK4, MTDH, TLR3) related to anoikis and EMT (Figure 2B). Their weighted coefficients are shown in Supplementary Table S1. Then, we used the model to calculate risk scores for WT patients in the training set and divided the patients into high-risk and low-risk groups based on the median risk score. To visually represent and validate the model’s accuracy, we conducted Kaplan–Meier (K-M) survival analysis for the high- and low-risk groups and calculated the area under the receiver operating characteristic (ROC) curve (AUC). The results showed that the survival rate of high-risk WT patients was significantly lower than that of the low-risk group (*p* < 0.0001) (Figure 2D), and the AUC values for 1 year, 3 years, and 5 years were around 0.8 (AUC = 0.79, 0.83, and 0.85, respectively) (Figure 2E), indicating the model’s high accuracy. Finally, we ranked the risk scores of each sample in the training set from high to low, resulting in risk score distribution plots, survival status plots, and a risk factors heatmap (Figure 2F). As shown in the figures, as patients’ risk scores increased, their overall survival (OS) times decreased significantly; the number of deaths increased, and the expression levels of most model genes exhibited an upregulation trend, confirming that they are risk genes. To assess the model’s robustness, we used the external independent dataset MP2PRT as a validation set. In keeping with the findings in the training set, the high-risk group had significantly lower OS than the low-risk group (*P* < 0.001) (Supplementary Figure S2C). In addition, the ROC curve, risk score distribution plots, survival status plots, and risk heatmap of the test cohort show the same trends as the training cohort, which demonstrates the applicability and robustness of the model across different datasets (Supplementary Figures S3D, E).

### 3.3 Correlation between risk scores and patients’ clinical characteristics

**FIGURE 3:**
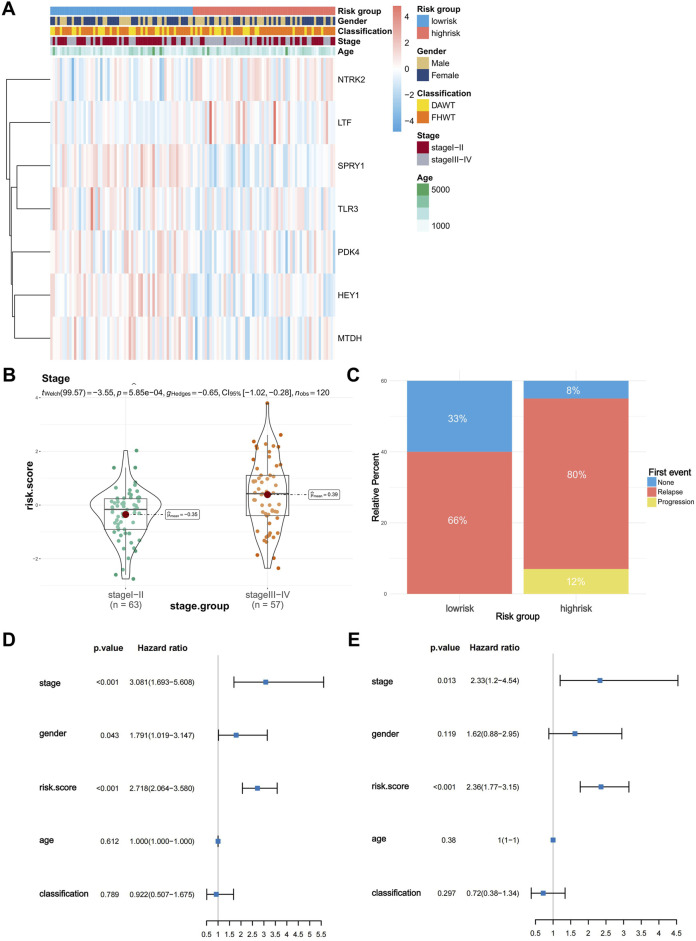
Risk score differences in clinical characteristics and independent predictive analysis of the prognostic model. **(A)** Heatmap of the correlation between model gene expression and clinical characteristics (stage, age, sex, classification). **(B)** Difference in risk scores between stage I-II and stage III-IV Wilms tumors. **(C)** First event information for the high-risk and low-risk groups. **(D,E)** Univariate and multivariate independent prognostic analyses of risk scores and clinical characteristics.

To explore the correlation between risk scores and clinical characteristics, we generated a heatmap showing the expression of the model genes used for risk grouping in WT patients and their correlation with clinical features (Figure 3A). Subsequently, we used the “ggstatsplot” package to compare the differences between different clinical feature groups. The results indicated that there was a significant difference in the different tumor stages, where stage III and IV patients had significantly higher risk scores than stage I and II patients (Figure 3B). However, there was no significant difference in risk scores among the patients in different age groups or in terms of histological classification and gender classification (Supplementary Figures S4A–C). Afterwards, we collected information about first events in the high- and low-risk groups and constructed a histogram (Figure 3C). It is evident that the probability of recurrence is significantly higher in the high-risk group, and all progressions occur within the high-risk group, while the proportion at which no first events occur is significantly higher in the low-risk group. We then conducted both univariate Cox (Figure 3D) and multivariate Cox (Figure 3E) independent prognostic analyses to determine whether the model we constructed could act independently of other clinical features. As shown in the figures, in the single factor Cox and multiple factor Cox analyses, the *P*-values of the risk score are both less than 0.001, indicating that the risk score can independently act as a prognostic factor without being affected by other clinical features; it therefore has better applicability and accuracy in clinical applications.

### 3.4 Construction and validation of the nomogram

**FIGURE 4:**
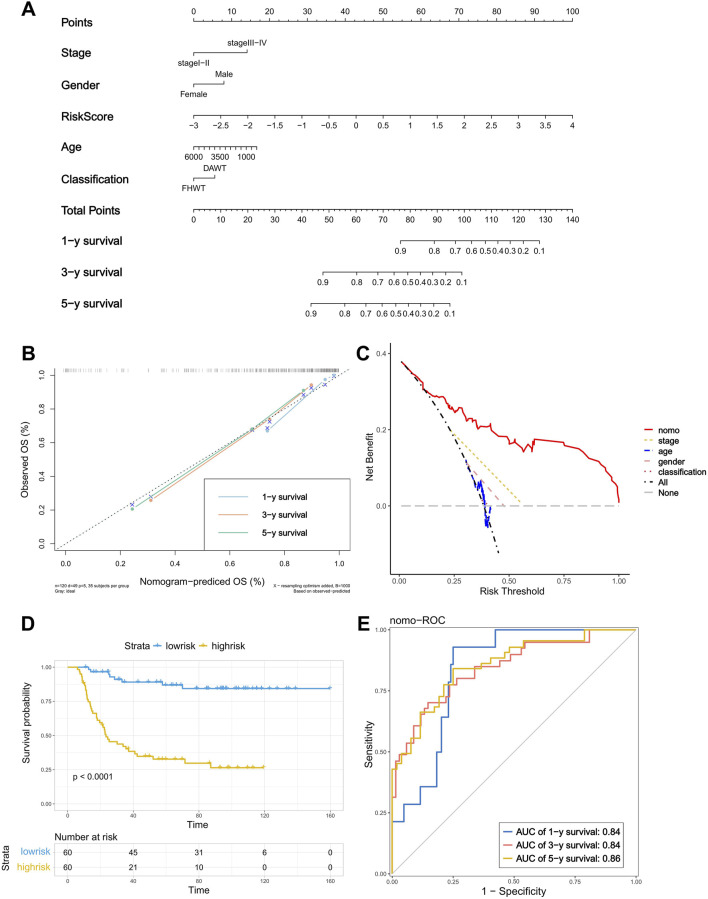
Construction and verification of the nomogram. **(A)** A nomogram based on risk score, age, gender, stage and classification. **(B)** Calibration plot of the nomogram used to predict the probability of OS at 1, 3, and 5 years. **(C)** DCA of the nomogram. **(D)** K–M curve of the nomogram (Log-rank test). **(E)** ROC curves of the nomogram for predicting 2-, 3-, and 5-year OS.

By combining the risk score with the stage and other clinical features, we constructed a nomogram to provide a quantitative method for the personalized prediction of patient clinical outcomes. As shown in the nomogram, each variable is represented by a line segment with markings indicating the range of possible values for that variable, and the length of the line segment reflects the contribution of that factor to the outcome events. We found that the risk score calculated by the model contributes more risk points compared to other clinical features (Figure 4A). In the calibration plot, the calibration curve was found to be close to the ideal line, indicating good consistency between prediction and observation (Figure 4B). The decision curve analysis (DCA) showed that the predictive performance of the nomogram was significantly higher than that of individual clinical features (Figure 4C). At the same time, the Kaplan–Meier analysis showed a significant difference in survival rates between the high- and low-risk groups, which, when divided by the median of total score calculated by the nomogram (Figure 4D) and the AUC of the nomogram’s ROC curve, exceeded 0.8 (AUC = 0.84, 0.84, and 0.86) (Figure 4E). These results demonstrate that the nomogram exhibits good predictive performance.

### 3.5 Gene mutation frequency of the prognostic model

To investigate the relationship between the tumor mutation burden (TMB) and the risk score, as well as their association with prognosis, we first used waterfall plots to visualize the mutation frequency and the types of the top 20 genes with the highest mutation rates in both the high-risk and low-risk groups (Supplementary Figure S5A). However, we found no significant differences between the two groups. Subsequently, we compared the TMB between the high-risk and low-risk groups and observed no significant differences (Supplementary Figure S5B).

### 3.6 Predicting the sensitivity of high- and low-risk groups to drug treatment and immunotherapy

**FIGURE 5:**
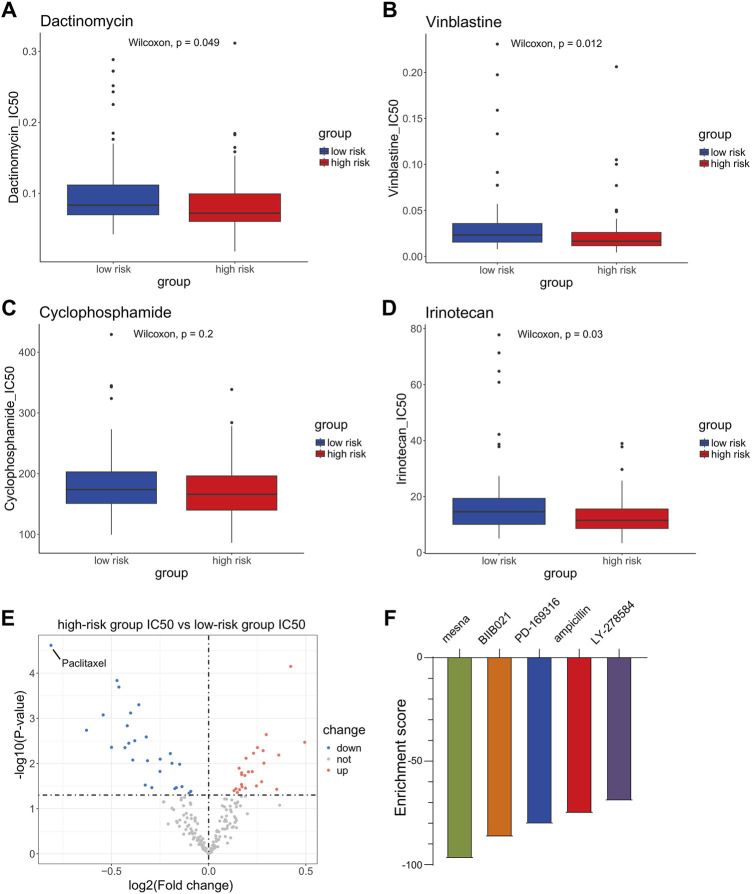
Sensitivity analysis of chemotherapeutic drugs and immunotherapy in high- and low-risk groups. **(A–D)** Difference in sensitivity for dactinomycin, vincristine, and cyclophosphamide and irinotecan between Wilms tumor (WT) patients in the high-risk group and the low-risk group. **(E)** Volcano plot of the difference in sensitivity to anticancer drugs between high-risk and low-risk WT patients. **(F)** Connectivity map (cMAP) analysis.

To further investigate the impact of anoikis and EMT on drug resistance in WT and the clinical implications of this signaling model, we downloaded the GDSC2 dataset as our training set and constructed a model using the “oncoPredict” package to assess the sensitivity of WT patients in different risk groups to drug treatments. The results showed that, among the chemotherapeutic drugs approved by the Food and Drug Administration (FDA) for WT treatment, patients in the high-risk group were more sensitive to treatment with dactinomycin and vincristine and irinotecan (Figures 5A–D). Based on this model’s prognostic assessment, high-risk patients may benefit from prioritizing treatment with these drugs. Then, we used a volcano plot to visualize the IC50 difference of the 198 compounds evaluated using the drug sensitivity model between the high-risk group and the low-risk group. The results showed that paclitaxel had the lowest log2(Fold change) value, indicating that, according to the drug sensitivity model, compared with the low-risk group, paclitaxel may show the most significant decrease in IC50 in the high-risk group (Figure 5E). In order to further screen for drugs suitable for high-risk patients, we used the edgeR algorithm to analyze the differentially expressed genes between the high-risk and low-risk groups; we inputted the most significant differences of 30 upregulated genes and 150 downregulated genes in the high-risk group into cMAP. The results showed that the top five drugs with the highest negative values were mesna, BIIB021, PD-169316, ampicillin, and LY-278584. Among them, only mesna had an enrichment score lower than −90, indicating that this drug represents a potential adjuvant therapy for high-risk patients (Figure 5F). Furthermore, we employed the tumor immune dysfunction and exclusion (TIDE) algorithm to assess the effectiveness of immune therapy for patients in different risk groups. Patients with higher TIDE scores are more likely to experience immune evasion, resulting in a reduced response to immune therapy. The results indicated that there were no significant differences in sensitivity to immune checkpoint inhibitor therapy between the high- and low-risk groups (Supplementary Figure S6).

### 3.7 Analysis of the relevant molecular mechanisms between high- and low-risk groups

**FIGURE 6:**
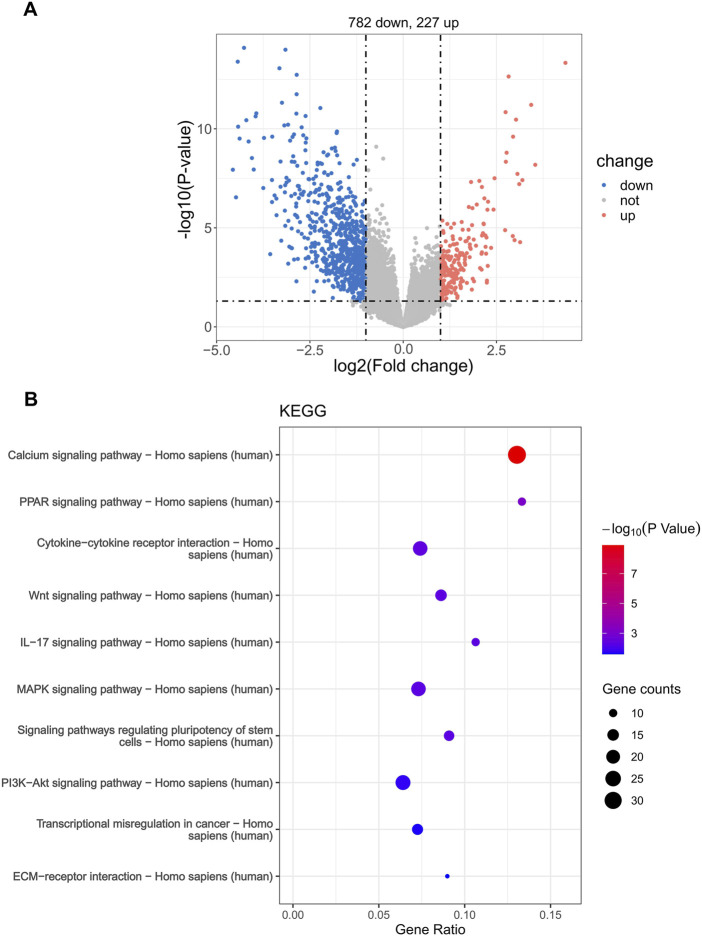
Enrichment analysis of the Kyoto Encyclopedia of Genes and Genomes (KEGG) differential pathway in the risk groups. **(A)** Volcano plot of DEGs between high- and low-risk groups. **(B)** KEGG pathway enrichment.

In order to explore the specific mechanisms that affect the risk scores of patients with WT, we used KEGG pathway enrichment analysis to find the pathway of differential expression between the two groups based on the differentially expressed genes in the high- and low-risk groups (782 downregulated genes and 227 upregulated genes) (Figure 6A). These differentially expressed genes were enriched in various cancer-related pathways, including cytokine−cytokine receptor interaction, PPAR, Wnt, IL−17, the MAPK signaling pathway, and transcriptional misregulation in cancer (Figure 6B). This indicates that the differentially expressed genes in the risk groups are related to the occurrence and development of WT.

### 3.8 Immune infiltration analysis and hub gene screening

**FIGURE 7:**
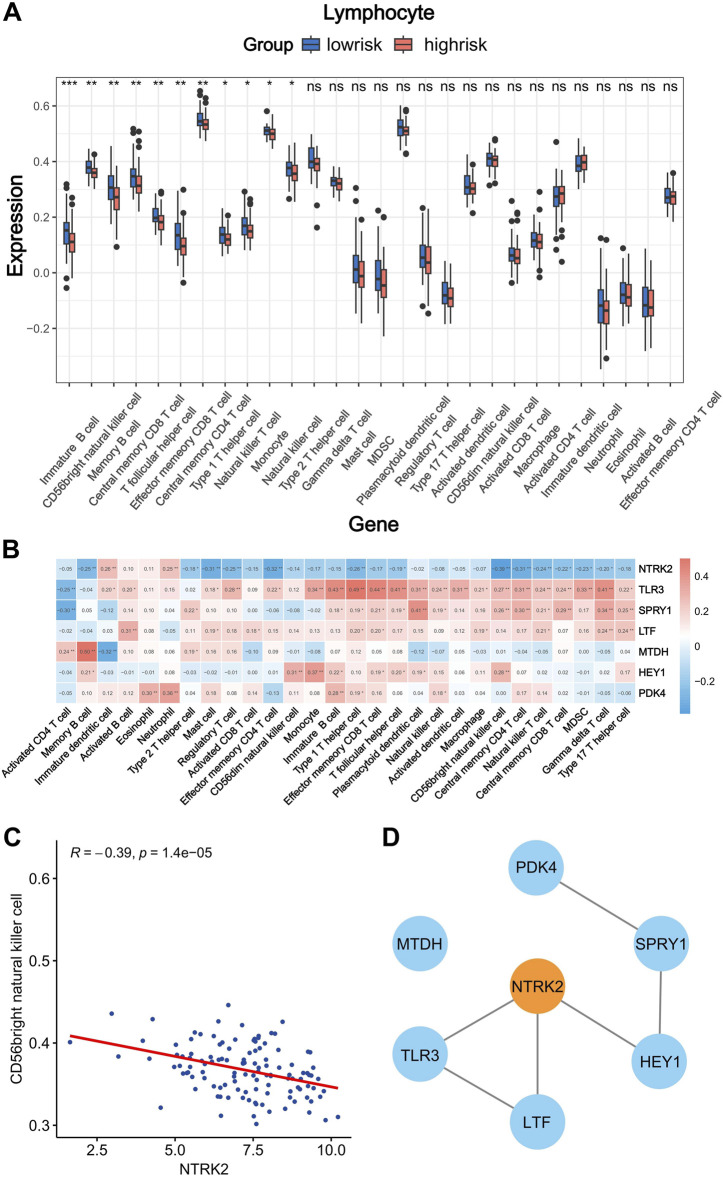
Correlation analysis of immune infiltration and screening of hub genes in the prognostic model. **(A)** Difference in immune cells infiltration abundance between high-and low-risk groups (^*^ *p* < 0.05; ^**^ *p* < 0.01; ^***^ *p* < 0.001). **(B)** Correlation between the expression of risk factors and the infiltration abundance of immune cells. Red, positive correlation; blue, negative correlation (^*^ *p* < 0.05; ^**^ *p* < 0.01). **(C)** Correlation between NTRK2 expression and CD56bright natural killer cell infiltration abundance. **(D)** Protein interaction network of risk factors.

Most of the previously enriched differential pathways of KEGG exhibit deep crosstalk with the tumor microenvironment; therefore, in order to further explore the relationship between the risk groups and the tumor microenvironment (TME), we employed the ssGSEA algorithm from the “GSVA” package. This was used to evaluate the differences in the abundance of various immune cell subtypes within the tumor microenvironment between the high- and low-risk groups. It is worth noting that, among the 28 types of infiltrating immune cells, there were 11 subtypes with significantly different abundance levels (Figure 7A). This proves that there are large differences in the abundance of immune cell infiltration in samples from the different risk groups. Moreover, we speculated that the expression of risk factors that constitute the prognostic model may affect the number of immune cells in the tumor microenvironment. Therefore, we calculated the correlation coefficients between the expression levels of key molecules and the abundance of TME-infiltrating cells in WT samples and then created a correlation heatmap with annotated correlation coefficients and *P* values (Figure 7B). The results demonstrated that risk factors were significantly correlated with most immune cells, and the correlation was mainly positive. It is worth noting that NTRK2 is significantly negatively correlated with various immune cell subpopulations, and overexpression of this gene may lead to an overall downregulation trend in immune cell infiltration in samples from the high-risk group. Additionally, the negative correlation between the NTRK2 gene and CD56^bright^ natural killer cells was the most significant, with correlation coefficients (R-values) reaching −0.39. (Figure 7C). Then, to select hub genes, we constructed a protein–protein interaction network for the model genes based on the STRING database and used the MCC algorithm to identify NTRK2 as the hub gene for this risk model; this gene is most widely associated with other genes and may dominate the model predictions (Figure 7D). Furthermore, it was found that NTRK2 is a risk gene and is upregulated in tumor tissues (Supplementary Figure S7). And we also demonstrated significant overexpression of NTRK2 in tumor tissue using the SPH cohort (Supplementary Figure S8).

### 3.9 Validation of NTRK2 expression levels in Wilms tumor cell lines and clinical samples

**FIGURE 8:**
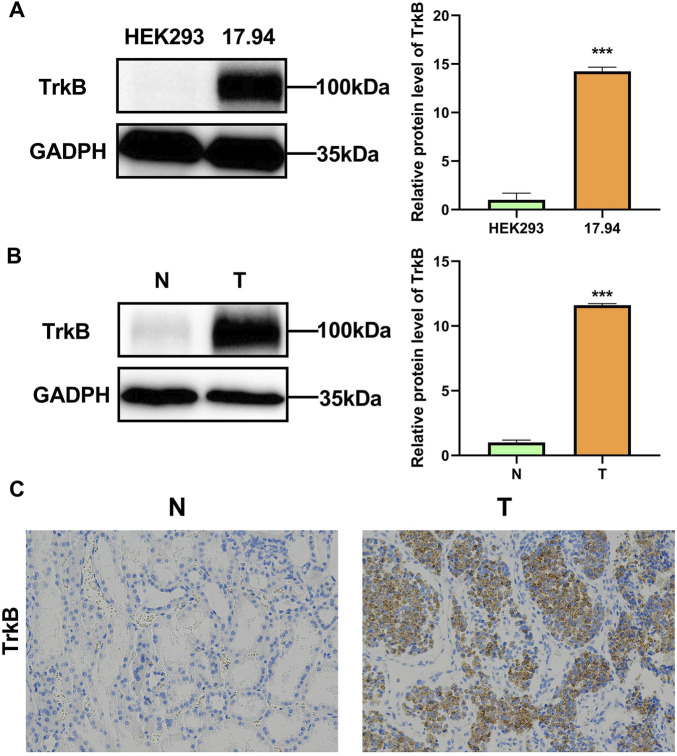
Verification of TrkB expression levels. **(A)** TrkB protein levels in the HEK293 and 17.94 cell lines were detected using WB. **(B)** TrkB protein levels in normal adjacent (N) tissues and Wilms tumor (T) tissues were detected using WB. **(C)** Expression levels of TrkB protein determined using IHC in normal adjacent (N) tissues and Wilms tumor (T) tissues (magnification: 100×).

To further clarify the difference in NTRK2 expression at the protein level between tumor tissues and adjacent tissues, we first detected the difference in the expression of the NTRK2-encoded protein TrkB between the Wilms tumor cell line 17.94 and the normal human embryonic kidney cell line HEK293 using Western blotting. The results showed that the expression level of TrkB in the 17.94 cell lines was significantly higher than that in HEK293 (Figure 8A). Subsequently, we used surgically collected Wilms tumor tissues and adjacent fresh frozen tissues and paraffin sections to detect the expression of the TrkB protein by WB and IHC, and the results showed that the TrkB protein was highly expressed in Wilms tumor tissues (Figures 8B, C).

### 3.10 The expression of NTRK2 promotes the proliferation, migration, and invasion of Wilms tumor cells

**FIGURE 9:**
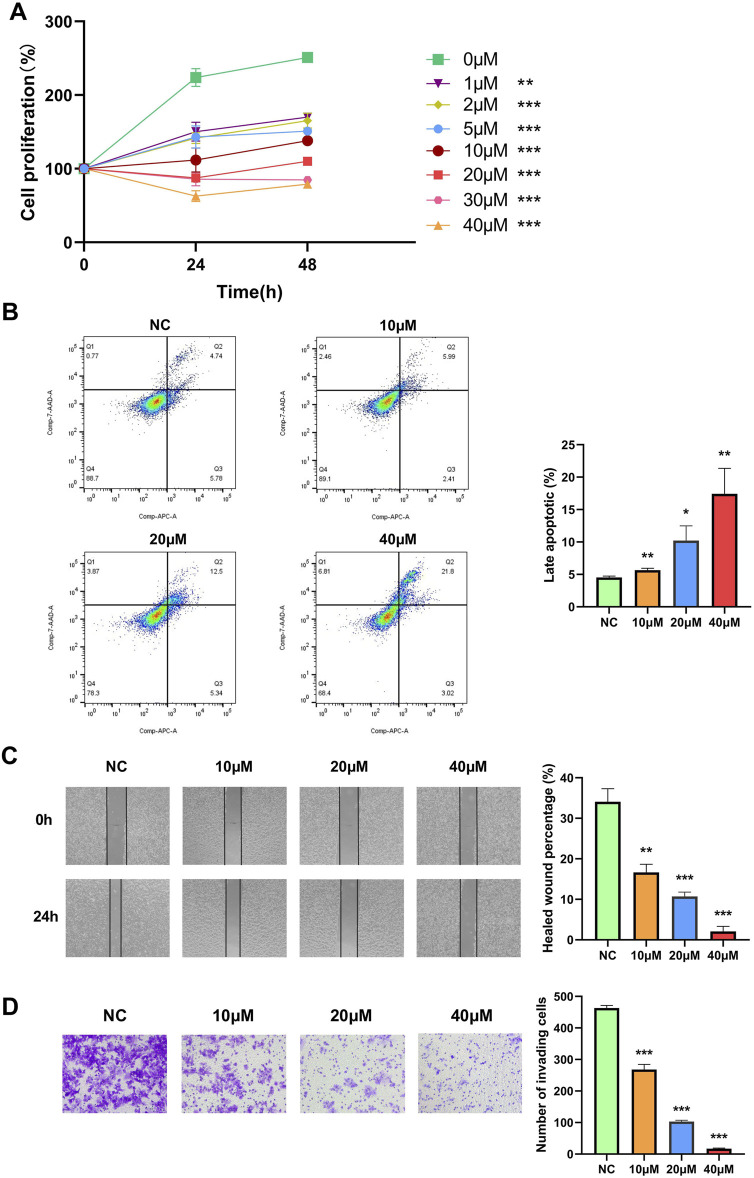
The selective TrkB antagonist ANA-12 inhibits the proliferation, migration, and invasion of the 17.94 cell line and promotes late apoptosis. **(A)** A CCK-8 cell proliferation assay was used to detect the proliferation ability of the 17.94 cell line. **(B)** Flow cytometry was performed to examine the late apoptosis of the 17.94 cell line. **(C)** The migration of the 17.94 cell line was detected using a wound healing assay (magnification: 10×). **(D)** A Transwell assay was used to detect the invasion of the 17.94 cell line (magnification: 20×). ^*^ *p* < 0.05; ^**^ *p* < 0.01; ^***^ *p* < 0.001.

To investigate the role of NTRK2 in WT cells, we treated 17.94 cells with the selective TrkB antagonist ANA-12 at different concentrations to target the downstream TrkB pathway. To evaluate the effects of TrkB inhibition on the viability of the 17.94 cells, we exposed the cells to varying concentrations of ANA-12 (0, 1, 2, 5, 10, 20, 30, and 40 μM) for different time periods (0, 24, and 48 h). A dose-dependent reduction in cell viability was observed in the cell lines, and increasing effects were observed for longer time periods (Figure 9A).

In addition, we exposed the cells to varying concentrations of ANA-12 (0, 10, 20, and 40 μM) for 24 h; the effects on apoptosis, cell migration, and invasion potential were then examined using flow cytometry, wound healing, and a Transwell assay. After 24 h, the flow cytometry showed that the use of ANA-12 increased the percentage of late apoptotic cells (Figure 9B). The 17.94 cell lines in the treated groups demonstrated significantly inhibited healing compared to the 0 μM group in the wound healing assay (Figure 9C). In addition, we demonstrated that ANA-12 inhibited cell invasion (Figure 9D). All the above results were dose dependent.

## 4 Discussion

In recent years, the significant role of anoikis and EMT in the migration and invasion of tumor cells has gained increasing attention. Both anoikis and EMT play important roles in distant metastasis, which severely limits the prognosis of WT patients (Liu et al., 2023; Cao et al., 2016). Therefore, it is highly important to explore the inherent crosstalk between a loss of nesting apoptosis and EMT and tumor metastasis. However, large-scale systematic analyses of and clinically robust models for anoikis and EMT-related genes in WT are still lacking. Additionally, further research is needed to screen hub biomarkers of anoikis and EMT in the occurrence, development, and resistance of WT.

In order to prove that there are pathological changes in the anoikis and EMT pathways of tumor cells in WT patients, we used the GSVA algorithm to evaluate the activation of these two pathways in tumor and normal tissues by calculating the pathway score of an anoikis-related gene set and an EMT-related gene set. We found that these two pathways were abnormally regulated in WT. Tumor tissue not only inhibits anoikis, but also excessively activates the biological function of EMT. Previous studies have shown that the inhibition of anoikis can prevent the apoptosis of cancer cells during distant metastasis, while the mesenchymal characteristics induced by EMT enable cancer cells to successfully complete the pathological processes of invasion and metastasis (Paoli et al., 2013; Nieto et al., 2016). We proved that, in WT, cancer cells may promote their own occurrence and metastasis by abnormally regulating ectopic apoptosis and EMT. Then, we established and validated a prognostic model based on ARG and ERG. We demonstrated its accuracy and robustness for both the training and validation sets. This prognostic model can also play an independent role in prognostic factors without being affected by other clinical features. This model consists of a total of seven genes: SPYR1, HEY1, LTF, NTRK2, PDK4, MTDH, and TLR3. In previous researches, several results suggest that Hey1 promotes the invasion and metastasis of melanoma cells by regulating the GRB2/PI3K/AKT pathway (Pu et al., 2021). The expression level of HEY1 in lung adenocarcinoma is upregulated. Patients with high HEY1 expression levels have poor prognosis after cisplatin therapy. HEY1 could regulate the cisplatin sensitivity of non-small-cell lung cancer (NSCLC) cells (Gao et al., 2022). Experiments have confirmed that the silencing of HEY1 expression can induce cisplatin resistance, and EMT changes occur during this process (Gao et al., 2022). The overexpression of LTF promotes the proliferation, migration, and invasion of osteosarcoma cells. LTF can serve as a prognostic biomarker for osteosarcoma (Liu et al., 2022). In Hu’s study, NTRK2 is an oncogene related to microRNA-22 regulation in human gastric cancer cell line (Hu et al., 2016). Research has verified that the upregulation of PDK4 expression enhances the ability of gastric carcinoma cells to proliferate, migrate, and invade (Zhang et al., 2022). The MTDH gene is amplified in human hepatocellular carcinoma (HCC) patients, and the overexpression of MTDH has been identified in a high percentage of both hepatitis-B-virus- and hepatitis-C-virus-positive HCC cases, suggesting its key role in regulating HCC (Sarkar, 2013). Bianchi, F.'s research shows that TLR3 expression in the early stage of NSCLC is associated with a good prognosis (Bianchi et al., 2020).

Next, we analyzed the correlation between the risk score and clinical characteristics of patients. The results showed that only different tumor stage groups exhibited a significant difference, with stage III and IV patients having significantly higher risk scores compared to stage I and II patients, which means the prognostic model can assist in the clinical evaluation of the tumor stage in patients with WT. However, there was no significant difference in risk scores among the different age groups, histological classifications, and gender classifications. We also examined the information on different first events in the high-risk and low-risk groups. It was evident that the probability of experiencing relapse or progression recurrence as the first event was significantly higher in the high-risk group. This further underscores the association between high risk levels and poor prognosis, demonstrating the accuracy of our risk scoring.

Subsequently, we explored the differences in the mutation burden between the high-risk and low-risk groups, as well as the association between the mutation burden and prognosis. However, no significant results were obtained in this regard. We then found that the model can provide some choices of treatment for WT patients. High-risk patients, as assessed using this model, may be prioritized for treatment with drugs such as dactinomycin, vincristine, and irinotecan. Meanwhile, there was no significant difference in the sensitivity to immune checkpoint inhibition therapy between the high-risk and low-risk groups. Then, we used a volcano plot to visualize the difference in the IC50 of the 198 compounds evaluated by this drug sensitivity model between the high-risk group and the low-risk group. The results showed that paclitaxel might be the most promising drug for the treatment of high-risk patients in this compound library. Previous studies initially demonstrated that paclitaxel combined with carboplatin or cisplatin can benefit heavily treated or adult WT patients (Ozaki et al., 2012; Ramanathan et al., 2000). In breast cancer, cisplatin and paclitaxel can jointly inhibit tumor growth and prevent tumor metastasis by blocking early EMT (Wang et al., 2021). These studies confirmed the reliability of the prediction and the potential of paclitaxel for further clinical treatment. In order to further expand the screening range of sensitive chemotherapeutic drugs in the high-risk group, we used cMAP to screen out the compound mesna, which may significantly reverse the expression of high-risk genes in the population of WT patients in this analysis. This drug is a uroprotective thiol agent, is routinely administered concomitantly with ifosfamide, and has almost eliminated ifosfamide-induced hemorrhagic cystitis, as well as reducing nephron toxicity (Dechant et al., 1991). Studies have shown that, as a component of combination regimens, it exhibits good efficacy in a variety of cancers, such as recurrent sarcomas, pulmonary pleomorphic carcinoma, epithelial ovarian carcinoma, and so on (Miser et al., 1987; Lee et al., 2018; Sutton, 1993). Therefore, it is speculated that the combined use of mesna during chemotherapy in WT patients can reduce chemotherapy-related side effects while also lowering patient risk scores and improving their prognosis.

To explore the specific mechanisms that affect the risk scores of patients with WT, we enriched the pathway of DEGs in the high- and low-risk groups based on the KEGG gene set. We found that they were primarily enriched in various cancer-related pathways, such as the PPAR signaling pathway, cytokine–cytokine receptor interactions, Wnt, MAPK, the IL-17 signaling pathway, and transcriptional misregulation in cancer. PPARs are metabolic regulators that participate in the regulation of glucose and lipid homeostasis, and there are three subtypes of PPARs that are encoded by distinct genes (Park and Kwak, 2012; Fucci et al., 2012). Previous studies have shown that the PPAR signaling pathway promotes proliferation and inhibits the apoptosis of cancer cells (Wang et al., 2022b). Cytokines are released in response to a diverse range of cellular stresses, including carcinogen-induced injury, infection, and inflammation. Whereas the effective containment of the injury promotes tissue repair, the failure to resolve it can lead to persistent cytokine production and to an exacerbation of tissue destruction. As such, host reactions to cellular stress can have an impact on multiple stages of cancer formation and progression (Dranoff, 2004). Aberrant Wnt signaling has been described as a key player in the initiation and/or maintenance and development of many cancers, due to its effect on the behavior of cancer stem cells (Duchartre et al., 2016). The MAPK signaling pathway is not only involved in regulating cellular biological functions, such as cell proliferation, cell differentiation, cell cycle regulation, cell apoptosis, and tissue formation, but is also related to tumor formation (Rubinfeld and Seger, 2005). Continuous activation of the MAPK signaling pathway can promote the transformation of normal cells into tumor cells, while the inhibition of the MAPK signaling pathway can restore tumor cells to a non-transformed state and can inhibit tumor growth (Sebolt-Leopold et al., 1999). In addition, interleukin-17 is closely related to immunity and inhibits the activity of NK cells, thereby promoting the occurrence and development of tumors (Wang et al., 2019). In summary, the pathway analysis revealed that the risk score is closely related to tumor occurrence, development, and immunity.

The abnormality of the immune system is an important factor in the occurrence and development of many cancers, and anoikis and EMT are closely related to it (Romeo et al., 2019; Liao et al., 2023). We analyzed the differences in the tumor microenvironment’s infiltration abundance of immune cells between the high-risk and low-risk groups using ssGSEA. We found that 11 out of 28 kinds of immune cells showed significant differences, and all of them were downregulated in the high-risk group. It is therefore proven that an immunosuppressive effect occurs in the high-risk group, which promotes the development of tumor and drug resistance, leading to a poor prognosis. Then, the correlation between the prognostic model genes and the immune system was analyzed; it was found that, except for NTRK2 and immune cell infiltration, which were mainly negatively correlated, the other model genes were positively correlated with most immune cells. Therefore, we speculate that the overexpression of NTRK2 may lead to an overall downregulation trend in immune cell infiltration in samples from the high-risk group. It is worth noting that NTRK2 showed the most significant negative correlation with CD56^bright^ natural killer cells whose activity was inhibited by the interleukin-17 signaling pathway, which was enriched in the previous KEGG analysis. Therefore, we speculate that the tumor tissue of high-risk patients may activate the IL-17 signaling pathway by up-regulating the expression of the NTRK2 gene, so as to inhibit the activity of CD56^bright^ natural killer cells to reduce its abundance of tumor infiltration, thus preventing its inhibition and the elimination of malignant tumor cells.

After an in-depth demonstration of the important crosstalk between the model genes and WT, we set out to further screen their hub gene as a biomarker with the potential to assist with clinical diagnosis and treatment. We used the STRING database to construct a protein–protein interaction network for the model genes and utilized the MCC algorithm to identify NTRK2 as the hub gene for this prognostic model. NTRK2 (Neurotrophic Receptor Tyrosine Kinase 2) is a protein coding gene, it encodes the protein TRKB, which is a member of neurotrophic tyrosine receptor kinase (NTRK) family (Rohrberg and Lassen, 2021). And this family also includes TrkA and TrkC, which bind to nerve growth factor (NGF) and neurotrophin-3 (NT-3) respectively (Solomon et al., 2019). TRKs can regulate cell proliferation, differentiation, and even apoptosis via the RAS/MAPKs, PI3K/AKT, and PLCγ pathways (Jiang et al., 2021). Gene fusions involving NTRK act as oncogenic drivers of a wide range of adult and pediatric tumors (Wu et al., 2014), and TRKs have become promising antitumor targets (Cocco et al., 2018). ANA-12, originally developed as an experimental antidepressant, selectively and efficiently inhibits TrkB by binding to both low- and high-affinity sites on the receptor extracellular domain, it showed direct and selective binding to TrkB without altering TrkA and TrkC functions (Cazorla et al., 2011). Since its inception, there have been quite a number of experimental studies to prove its effectiveness. ANA-12 was identified as a selective NTRK2 antagonist, which has been reported to reduce chronic pain in different experimental models (Tillu et al., 2015; Liu et al., 2018). Furthermore, ANA-12 has been used to target NTRK2 in studies of medulloblastoma (Thomaz et al., 2019), gliomas (Pinheiro et al., 2017), leukemia (Polakowski et al., 2014), lung adenocarcinoma metastasis (Sinkevicius et al., 2014), lymphoid tissue neovascularization (Dalton et al., 2015) and endometriosis (Lee et al., 2021), etc. Experimental validation was performed on the hub gene. WB and IHC displayed an upward trend of TRKB protein expression in Wilms tumors, which is consistent with the results of a previous analysis of the public database. Finally, in order to further explore the effect of NTRK2 inhibition on the function of WT cells, we used *in vitro* experiments to clearly demonstrate that the downregulation of NTRK2 could inhibit the proliferation, migration, and invasion capabilities of the WT cell line 17.94 while increasing the percentage of late apoptotic cells. This proved the inherent potential of NTRK2 to act as a prognostic biomarker and a drug target for patients with WT.

However, this study has certain limitations. We did not conduct a detailed analysis of the interactions between key molecules and the specific molecular mechanisms by which anoikis and EMT impact WT.

## 5 Conclusion

This study systematically demonstrated the importance of the misalignment and crosstalk of the anoikis pathway and the EMT pathway in WT patients in tumorigenesis and development. We constructed a prognostic model composed of seven risk factors of ARGs and ERGs to predict the prognosis of WT patients and verified its reliability and robustness using a training cohort and a test cohort. In addition, we discussed the predictive effect of the risk model on the sensitivity of commonly used chemotherapeutic drugs and screened out potential drugs that might improve the clinical treatment of patients in the high-risk group. Then, using KEGG enrichment, we associated the risk model with TME and analyzed the differential genes between the high- and low-risk groups to explore the regulatory mechanism behind them. Finally, we screened the hub genes of the prognostic model through the STRING database and verified their inherent potential as prognostic biomarkers and drug targets using clinical samples and *in vitro* experiments.

## Data Availability

The datasets presented in this study can be found in online repositories. The names of the repository/repositories and accession number(s) can be found in the article/Supplementary Material.
