# Supplementary material for: The synergistic effects of anoikis-related genes and EMT-related genes in the prognostic prediction of Wilms tumor
Source: Front Mol Biosci. 2024 Sep 16;11:1469775. doi: 10.3389/fmolb.2024.1469775 (PMC11439783; doi:10.3389/fmolb.2024.1469775)
Supplement: Supplementary file 1 [file Table1.DOCX]

**Supplementary Table S1** The weighted coefficients of prognostic genes .

| gene | coef |
| --- | --- |
| NTRK2 | 0.2539 |
| SPRY1 | -0.8603 |
| HEY1 | -0.2904 |
| LTF | 1.3180 |
| PDK4 | -0.3391 |
| MTDH | -0.8709 |
| TLR3 | -0.3310 |
