# Supplementary material for: The synergistic effects of anoikis-related genes and EMT-related genes in the prognostic prediction of Wilms tumor
Source: Front Mol Biosci. 2024 Sep 16;11:1469775. doi: 10.3389/fmolb.2024.1469775 (PMC11439783; doi:10.3389/fmolb.2024.1469775)
Supplement: Supplementary file 2 [file Image1.pdf]

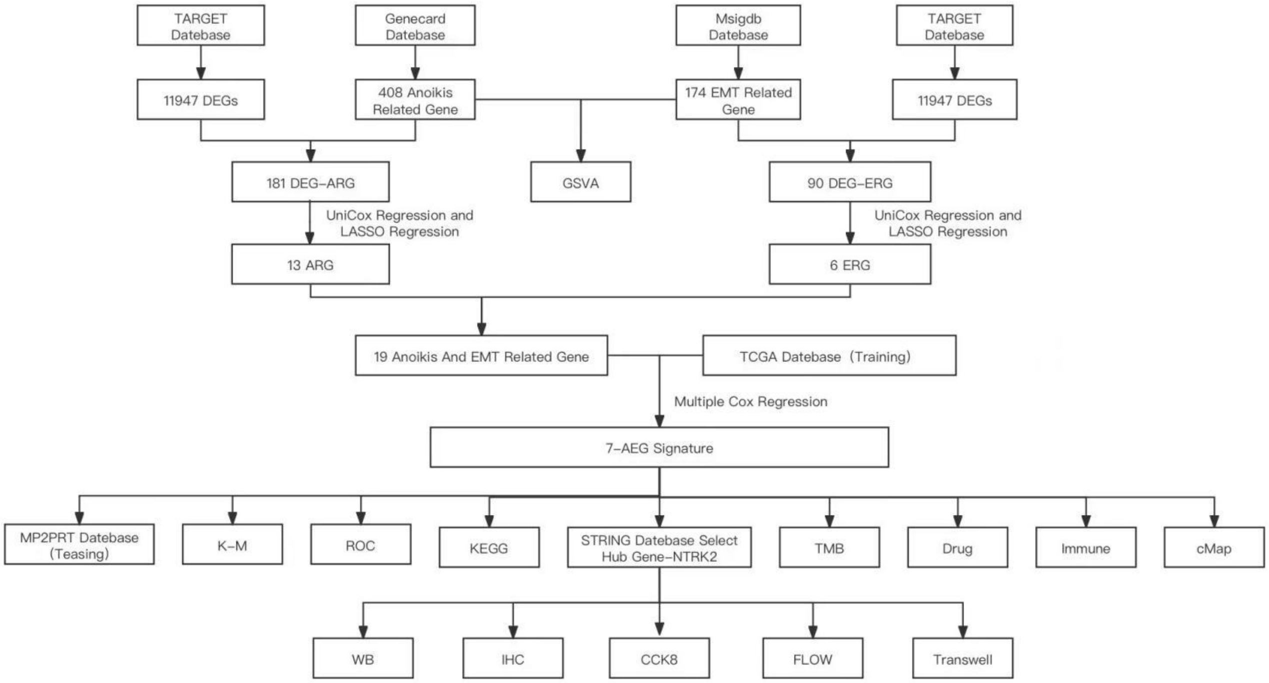

Supplementary Figure S1 The flow diagram of this research.

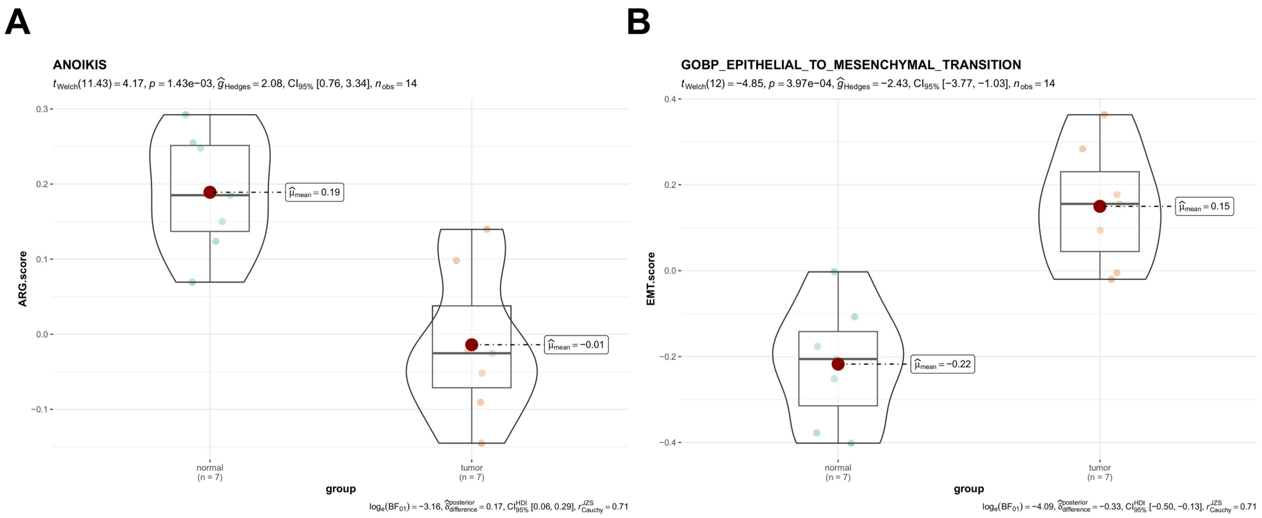

Supplementary Figure S2 (A, B) Difference in the anoikis and epithelial-mesenchymal transition (EMT) pathway score between normal and tumor tissues from the SPH cohort.

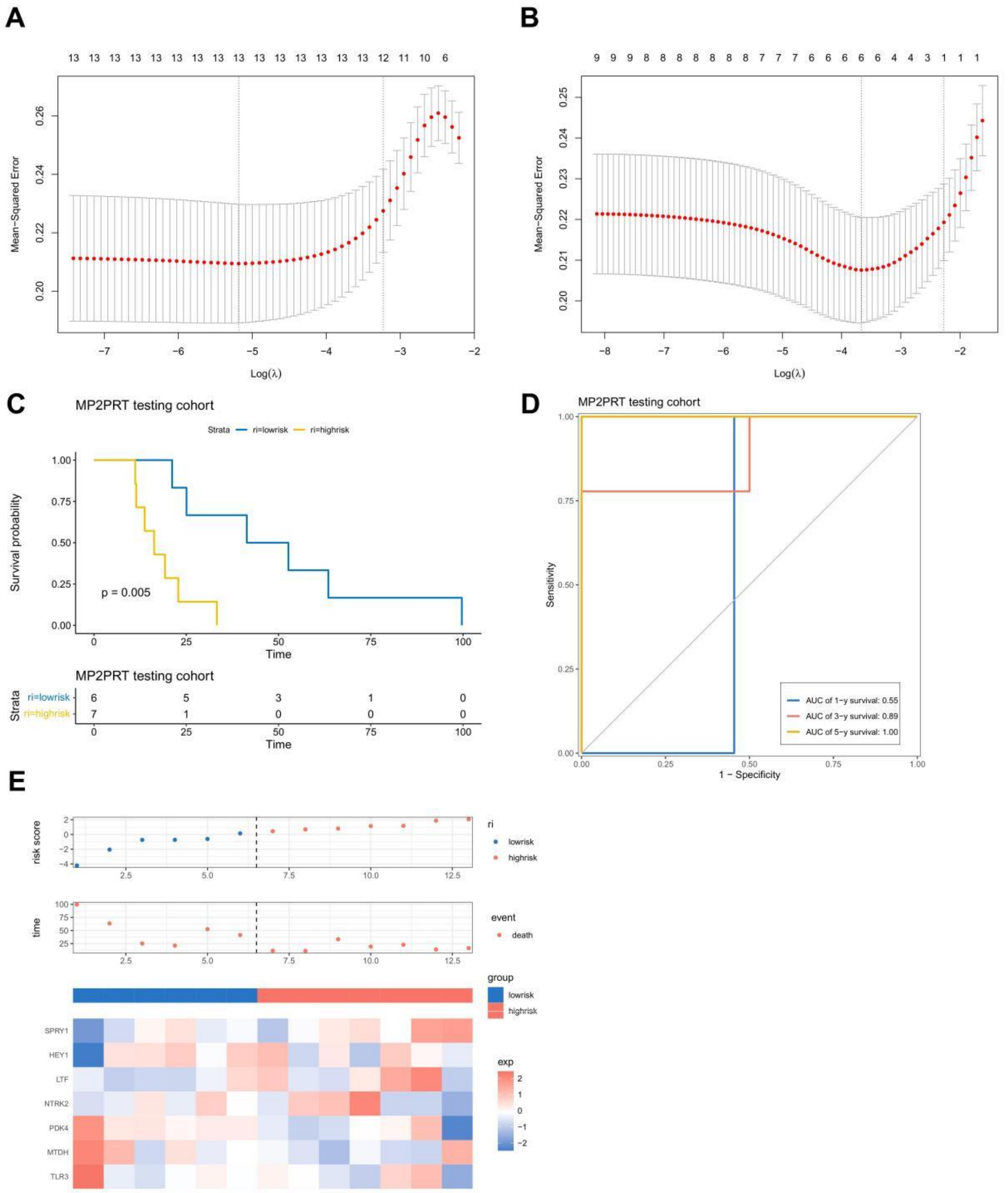

Supplementary Figure S3 (A, B) LASSO regression analysis log lambda of ARG and ERG. (C) K-M curve of the prognosis signature in the MP2PRT testing cohort (Log-rank test). (D) ROC curves of the prognostic model for predicting the 1-, 3-, and 5-year OS times in the testing cohort. (E) Risk score distribution plots, survival status plots, and risk heatmap of the MP2PRT test cohort.

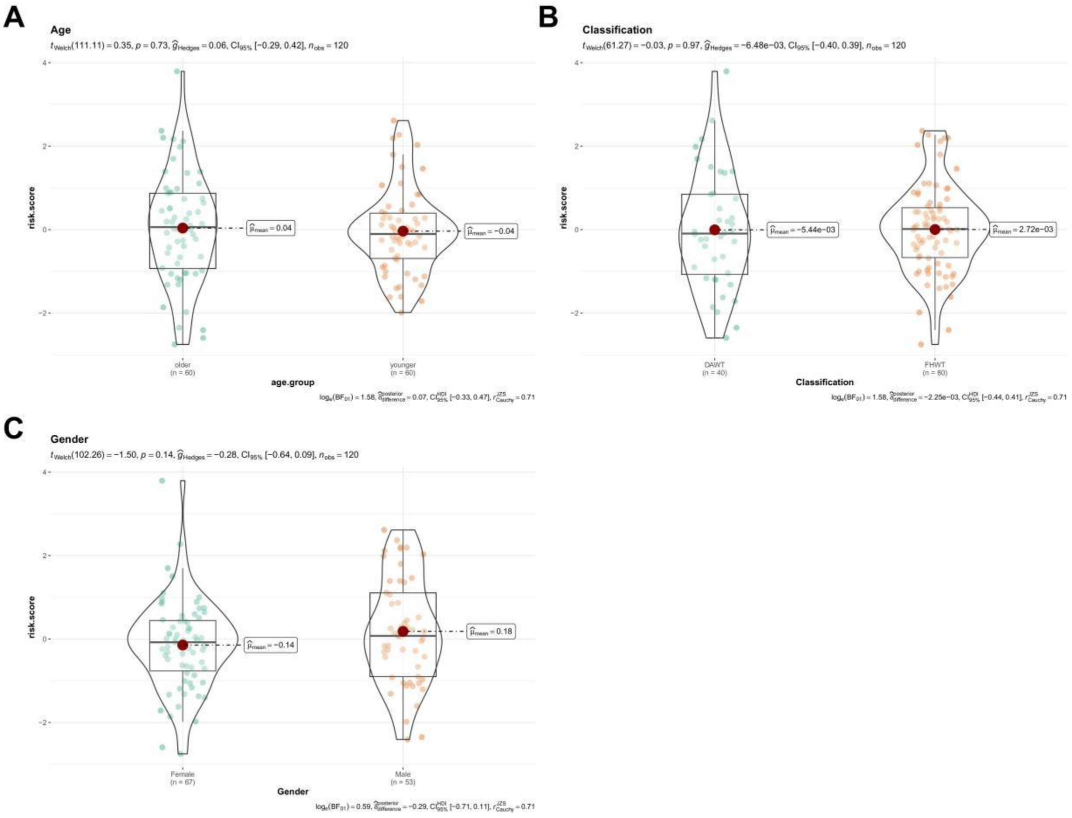

Supplementary Figure S4 (A) The difference of risk score among age groups. (B) The difference of risk score among classification groups. (C) The difference of risk score among different genders.

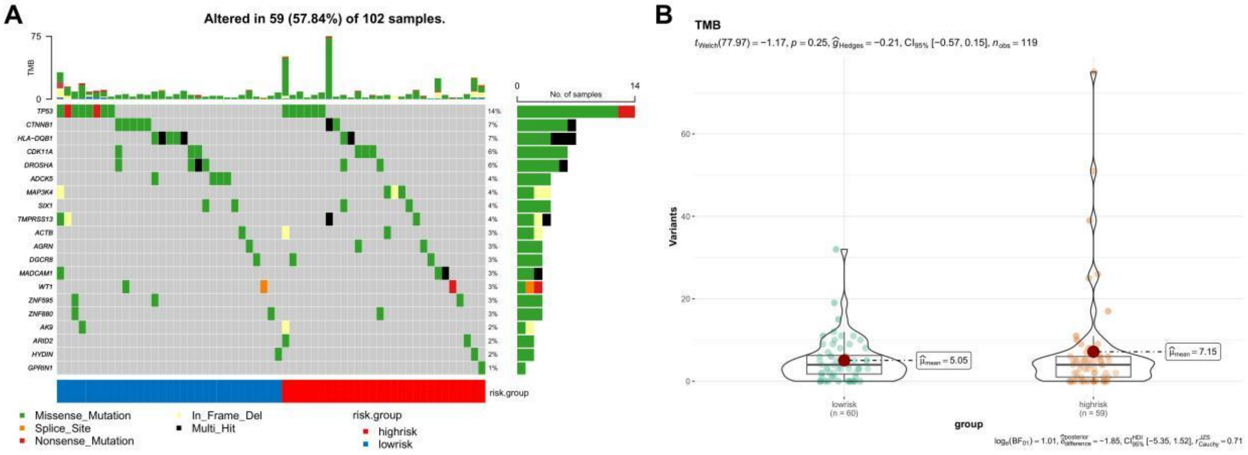

Supplementary Figure S5 (A) Top-20 gene mutation frequency in high and low risk groups. (B) The difference in the tumor mutation burden (TMB) between the high-risk and low-risk groups.

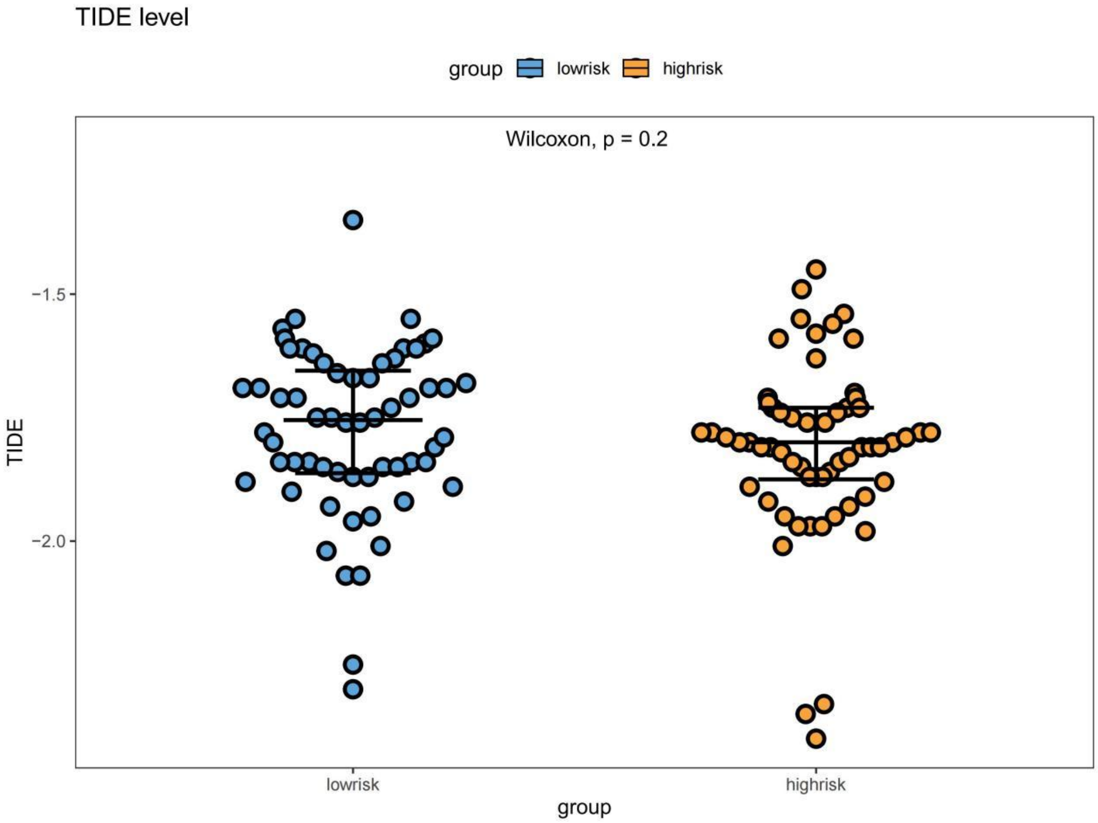

Supplementary Figure S6 Difference of TIDE scores between high risk group and low risk group.

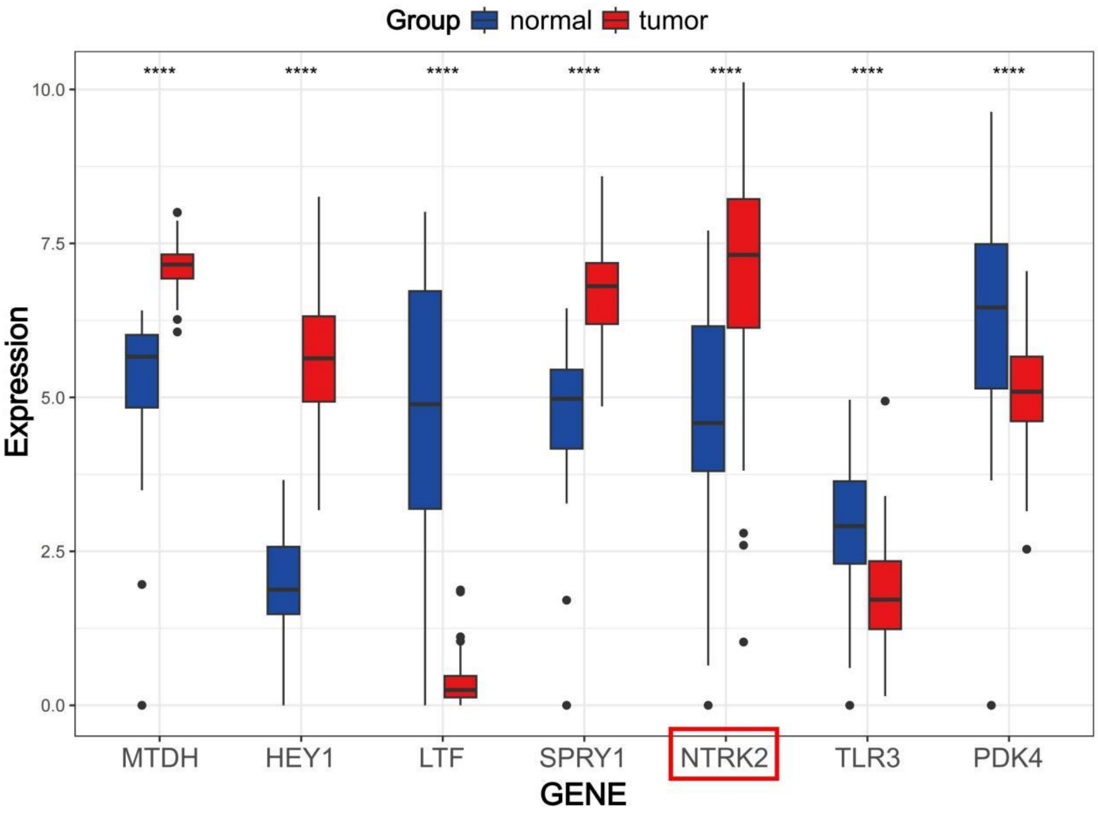

Supplementary Figure S7 Differential expression of risk factors between tumor and normal tissues in training cohort.

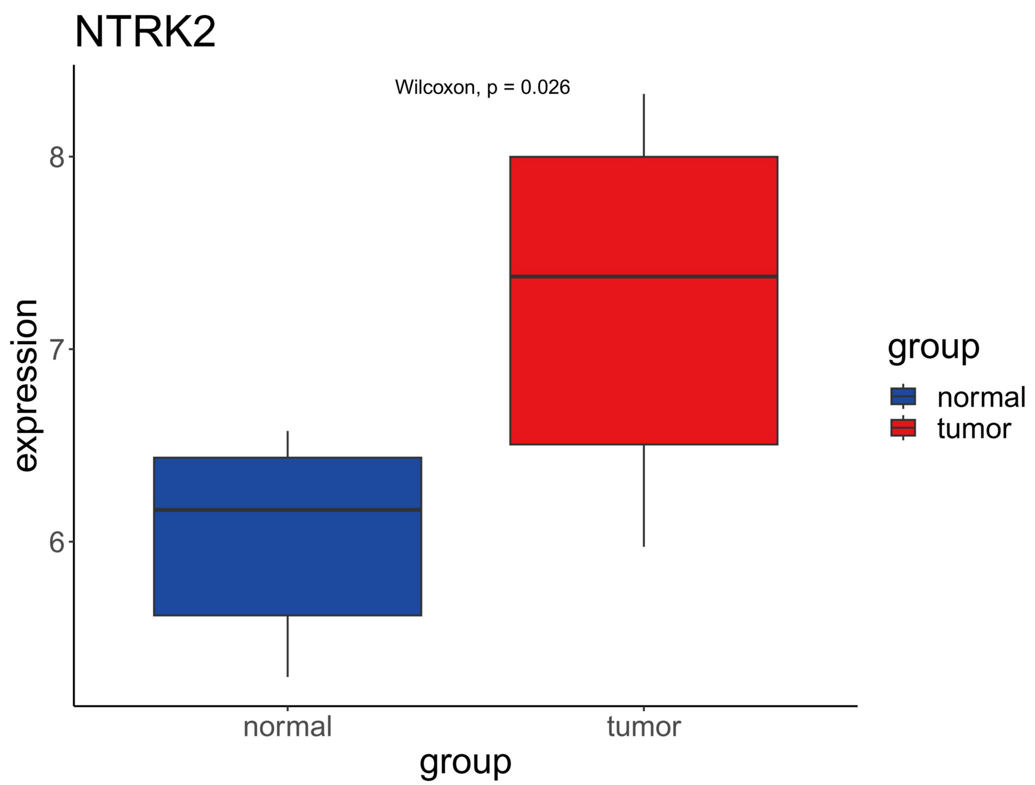

Supplementary Figure S8 Differential expression of NTRK2 between tumor and normal tissues in SPH cohort.
